# Impact of ^90^Y PET gradient-based tumor segmentation on voxel-level dosimetry in liver radioembolization

**DOI:** 10.1186/s40658-018-0230-y

**Published:** 2018-11-30

**Authors:** Justin K. Mikell, Ravi K. Kaza, Peter L. Roberson, Kelly C. Younge, Ravi N. Srinivasa, Bill S. Majdalany, Kyle C. Cuneo, Dawn Owen, Theresa Devasia, Matthew J. Schipper, Yuni K. Dewaraja

**Affiliations:** 1 Department of Radiation Oncology, University of Michigan, Ann Arbor, MI 48109 USA; 2 Department of Radiology, University of Michigan Medical School, Ann Arbor, MI USA

**Keywords:** ^90^Y, Tumor segmentation, ^90^Y PET, Radioembolization, Dosimetry, Auto-segmentation, Gradient-based segmentation

## Abstract

**Background:**

The purpose was to validate ^90^Y PET gradient-based tumor segmentation in phantoms and to evaluate the impact of the segmentation method on reported tumor absorbed dose (AD) and biological effective dose (BED) in ^90^Y microsphere radioembolization (RE) patients. A semi-automated gradient-based method was applied to phantoms and patient tumors on the ^90^Y PET with the initial bounding volume for gradient detection determined from a registered diagnostic CT or MR; this PET-based segmentation (PS) was compared with radiologist-defined morphologic segmentation (MS) on CT or MRI. AD and BED volume histogram metrics (D90, D70, mean) were calculated using both segmentations and concordance/correlations were investigated. Spatial concordance was assessed using Dice similarity coefficient (DSC) and mean distance to agreement (MDA). PS was repeated to assess intra-observer variability.

**Results:**

In phantoms, PS demonstrated high accuracy in lesion volumes (within 15%), AD metrics (within 11%), high spatial concordance relative to morphologic segmentation (DSC > 0.86 and MDA < 1.5 mm), and low intra-observer variability (DSC > 0.99, MDA < 0.2 mm, AD/BED metrics within 2%). For patients (58 lesions), spatial concordance between PS and MS was degraded compared to in-phantom (average DSC = 0.54, average MDA = 4.8 mm); the average mean tumor AD was 226 ± 153 and 197 ± 138 Gy, respectively for PS and MS. For patient AD metrics, the best Pearson correlation (*r*) and concordance correlation coefficient (ccc) between segmentation methods was found for mean AD (*r* = 0.94, ccc = 0.92), but worsened as the metric approached the minimum dose (for D90, *r* = 0.77, ccc = 0.69); BED metrics exhibited a similar trend. Patient PS showed low intra-observer variability (average DSC = 0.81, average MDA = 2.2 mm, average AD/BED metrics within 3.0%).

**Conclusions:**

^90^Y PET gradient-based segmentation led to accurate/robust results in phantoms, and showed high concordance with MS for reporting mean tumor AD/BED in patients. However, tumor coverage metrics such as D90 exhibited worse concordance between segmentation methods, highlighting the need to standardize segmentation methods when reporting AD/BED metrics from post-therapy ^90^Y PET. Estimated differences in reported AD/BED metrics due to segmentation method will be useful for interpreting RE dosimetry results in the literature including tumor response data.

**Electronic supplementary material:**

The online version of this article (10.1186/s40658-018-0230-y) contains supplementary material, which is available to authorized users.

## Background

Transarterial radioembolization (RE) with ^90^Y loaded microspheres is a valuable treatment option for unresectable hepatocellular carcinoma (HCC) and liver metastases [1]. Reported absorbed doses (AD) and biological effective doses (BED) for tumor response in RE are quite variable [2, 3]. This variability stems from several sources including microsphere device (glass or resin), tumor type, response metric, and dosimetry model used. Furthermore, tumor segmentation methodology is not standardized and end-user-specific details are often lacking in the literature. Tumor segmentation method, specifically morphological or functional, has been identified as a factor leading to significant differences in RE dosimetry [3].

Both an estimate of the dose distribution and segmentation is required for reporting tumor AD/BED metrics. As reviewed by Smits et al. [4], dose distributions in RE have been estimated from pre-treatment ^99m^TcMAA SPECT as well as from post-therapy ^90^Y imaging. It has been documented that MAA distributions are not always concordant or predictive of ^90^Y microsphere distributions [5], thus for establishing dose–response, post-therapy ^90^Y imaging is preferred. Some RE studies have performed tumor segmentation on diagnostic contrast computed tomography (CT) or magnetic resonance imaging (MRI) scans [2, 6–9] while others have used emission-driven segmentation on FDG PET [10, 11] or ^99m^TcMAA SPECT [12–14] with a focus on threshold-based delineation of tumors. A phantom study by Garin et al. [15] showed that thresholding of MAA SPECT alone had errors from 20 to 210% on average for two observers; these average errors were reduced to below 10% when guided by CT of the SPECT/CT. Chiesa et al. [14] compared MAA SPECT thresholding with CT manual segmentation and found the median absorbed doses for responding lesions to be 521 and 339 Gy, respectively. The scarcity of studies comparing segmentation methods and the reported large AD differences summarized in [2] demonstrate the need for studies comparing segmentation methods in RE and their effect on reported AD/BED values.

Although threshold-based segmentation is practical to implement, it is not robust under different imaging conditions [16]. The optimal threshold level that gives the best correspondence between the segmented volumes and ground truth has been shown to be highly dependent on target size, uptake heterogeneity, tumor-to-background ratio (TBR), and reconstruction method. An alternative emission tomography-based segmentation is the gradient-based method, which determines edges of a target based on changes in image intensity values at the boundary of the target. A phantom study has demonstrated higher accuracy with gradient-based segmentation compared with threshold based segmentation in FDG PET [17]**.** Gradient-based methods are semi-automated, require minimal user interaction, and overcome limitations of simple threshold-based methods. Furthermore, as highlighted in a recent AAPM task group report on auto-segmentation of PET, a primary advantage of gradient-based methods over thresholding is that the activity distribution can be non-uniform within the tumor and background [16]. This is particularly relevant in radioembolization where tumors may not be fully perfused and microsphere deposition is highly non-uniform due to clustering of microspheres [18]. A clinical implementation of the gradient-based method (PETEdge, MIM Inc., Cleveland, OH) has shown high accuracy and improved reproducibility on FDG PET/CT in lung tumors [19] and solid tumors [20]. Conclusions drawn from segmentation studies with ^18^F FDG PET may not be applicable to ^90^Y PET as microspheres are physically trapped in microvasculature, not metabolized into cells like FDG. Additionally, ^90^Y PET images are much noisier than FDG PET images due to the low positron yield and high fraction of random coincidence events [21]. However, to the best of our knowledge, the gradient-based method or other emission tomography-based segmentation methods have not been evaluated for ^90^Y PET.

The primary goal of this work was to quantify differences in ^90^Y tumor AD/BED estimates when using PET-based segmentation (PS) and morphologic segmentation (MS); specifically, a commercially available semi-automated gradient-based PS on post-therapy ^90^Y PET/CT was compared with manually delineated MS on CT or MRI. In addition to AD/BED metrics, differences in volume and spatial concordance between the two methods as well as intra-observer variation of the PS were quantified in both phantoms and patients.

## Methods

### ^90^Y PET/CT imaging

^90^Y PET/CT phantom and patient images were acquired with a Siemens Biograph mCT (Siemens Molecular Imaging, Hoffman Estates, IL); Phantom and patient PET data were reconstructed with Siemens 3D-OSEM software using the following parameters that were chosen based on a previous [22] phantom evaluation of contrast, quantification, and noise: resolution recovery, time-of-flight, 1 iteration 21 subsets, 5-mm Gaussian post-filter. The PET matrix size was 200 × 200 with a pixel size 4.07 × 4.07 mm and a slice thickness of 3 mm. The low-dose CT was acquired with tube voltage and current of 130 kVp and 80 mAs. The CT matrix size was 512 × 512 with a pixel size of 0.97 × 0.97 mm and a slice thickness of 2 mm. Identical reconstruction parameters were used for phantoms and patients.

### Phantoms

**Fig. 1:**
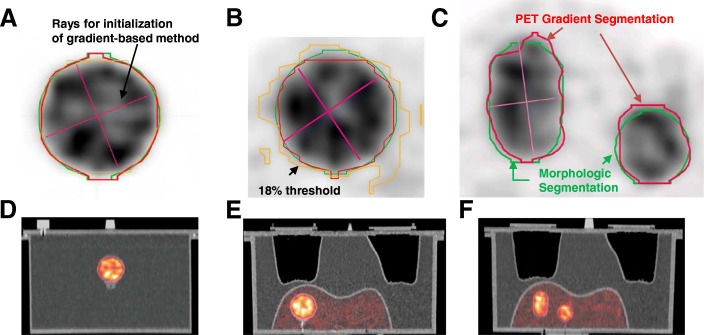
^90^Y PET (top) and fused PET/CT (bottom) corresponding to the phantom studies showing MS (green) and PS (red). Example rays from PETEdge tool are also shown. A threshold of 18% (orange) provided the best volume estimate (61.8 cc) to the MS in (**a**) (cold background), while the same 18% threshold overestimated the sphere volume (102.4 cc) in (**b)** (warm background). A 32% threshold (not shown) gave the best volume estimate (61.7 cc) for the MS in (**b**). Segmentation overlays on the zoomed in PET component in (**a**),(**b**),(**c**) corresponding to the the fused PET/CT in (**d**), (**e**), and (**f**), respectively

Three ^90^Y liver phantom studies were performed (Fig. 1). The first consisted of a 60 cm^3^ “hot” sphere positioned at the center of a “cold” water-filled phantom. The next two studies were performed with a liver/lung torso phantom (Data Spectrum Corporation, Durham, NC, USA) modified to include “tumor” inserts in the 1200 cm^3^ liver mimicking conditions following ^90^Y RE. In one case, a single 60 cm^3^ “hot” sphere was positioned in the “warm” liver with a TBR of 5:1, while in the next case two spheres (16 and 8 cm^3^) and an ellipsoid (29 cm^3^) were positioned in the “warm” liver TBRs of 5.1, 6.2, and 5.5 for the 8, 16, and 29 cm^3^ targets, respectively. The volumes 1200, 60, 16, and 8 cm^3^ are nominal values; in this work, we take the CT-based segmentation as the true volume. The total ^90^Y activity in the liver (3.0 GBq) and acquisition time (25 min) for the latter experiment was selected to achieve a count/noise-level typical for patient imaging following RE with glass microspheres.

To illustrate limitations with thresholding, the threshold that best estimated the 60 cm^3^ sphere’s CT-defined volume in both cold and warm backgrounds was manually found by adjusting the threshold level. During the threshold procedure, a spherical region was set to encompass each target with an approximately 1 cm margin as seen on CT. The purpose of these spherical regions/masks was to bound the threshold volume to exclude other targets when applying a threshold. The found threshold was then applied to the sphere in the other background to estimate volume differences.

### Patients

Imaging data of 18 patients (21 ^90^Y PET/CT scans as 3 patients had 2 treatments) treated with ^90^Y glass microspheres (Therasphere; BTG International Ltd., Ottawa, Canada) at our institution were retrospectively analyzed. The study was approved by the University of Michigan Institutional Review Board and all subjects signed an informed consent for ^90^Y PET/CT imaging as part of an ongoing research study. Administered activities were determined following standard clinical protocol, which is based on recommendations in the package insert. As discussed below, we did not include tumors < 2 cm^3^, thus Table 1 summarizes characteristics for the remaining 58 lesions in 15 patients (17 scans) included in our analysis. Post-therapy ^90^Y PET/CT imaging consisted of 25–30 min continuous bed motion acquisitions localized over the liver performed a few hours after infusing microspheres.

**Table 1:** Summary of analyzed patient and lesion characteristics

Characteristic	Patients (radioembolizations) or Average,median, (range)
HCC	4 (4)^a^
Cholangiocarcinoma	1 (1)
Liver metastases^b^	10 (12)
Total	15 (17^c^)
Elapsed time between baseline segmentation scan and 90Y PET/CT (days)	57, 49, (0 to 178)
Elapsed time between administration and ^90^Y PET/CT (min)	139, 144, (44 to 211)
Administered activity (GBq)	2.9, 3.2, (0.6 to 5.8)
Treated volume when determining administered activity (cm^3^)	1167, 1138, (190 to 2285)
MAA Lung shunt (%)	5.5, 4.4, (1.2 to 17.1)
Lesions per scan (*N* = 58)	3.4, 3 (1 to 9)
Lesion volume (morphologic) (cm^3^)	48.4, 11.4 (2.0 to 818)

^a^All 4 had cirrhotic livers

^b^Includes neuroendocrine, colorectal, pancreatic, melanoma, and adrenal disease

^c^12 right lobe and 5 left lobe

### Morphologically driven segmentation

In phantom studies, volumes of interest (VOI) consisted of manual delineation on axial slices of the non-contrast CT of the PET/CT by a medical physicist (JM). Lesion outlines were well-visualized on CT as evident in Fig. 1.

**Fig. 2:**
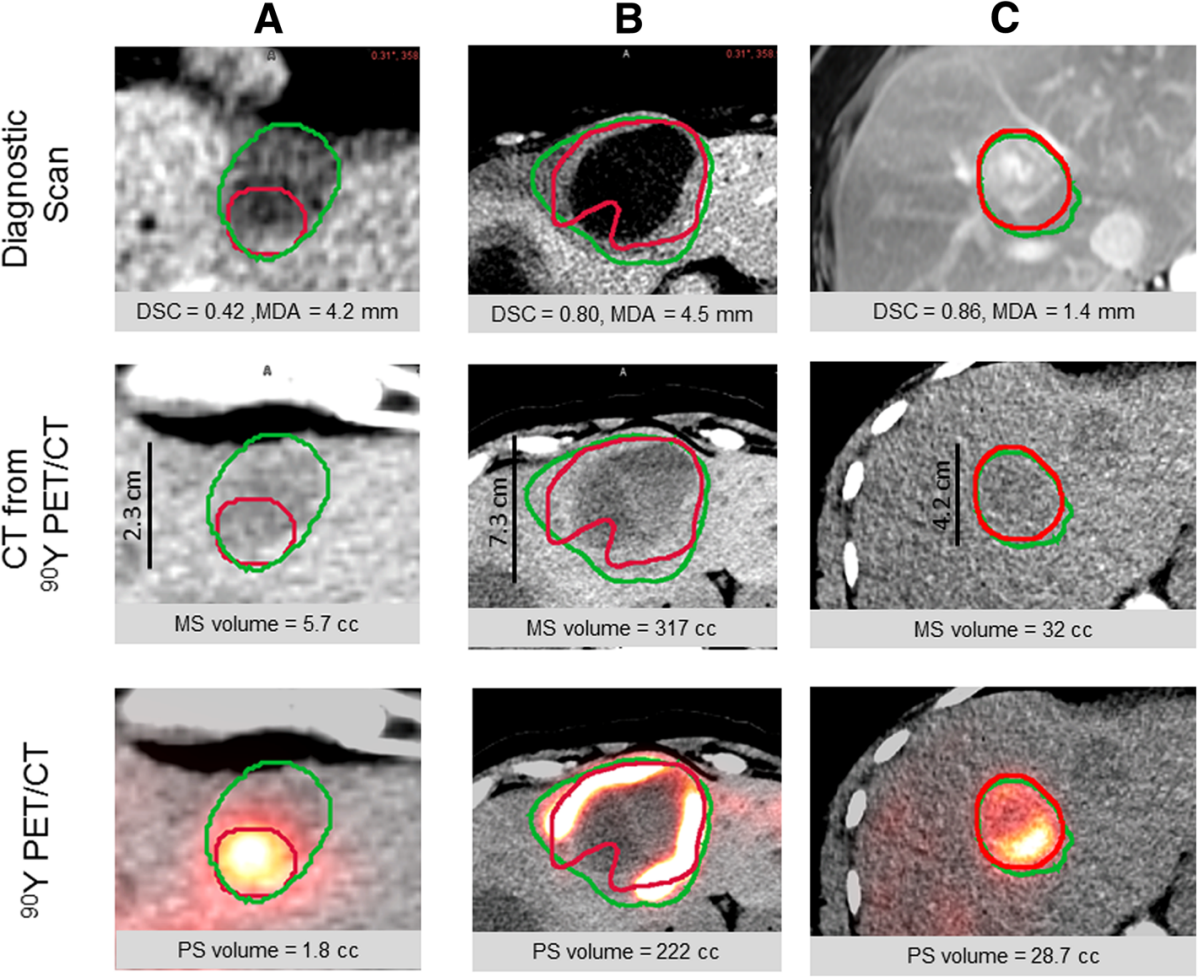
Three examples (**a**–**c**) showing PET-based (red) and morphologic (green) segmentations on a single axial slice. Note the scale is different across the three cases and the metrics were evaluated over the full 3D extent of the VOIs

In patient studies, lesions were usually not well-visualized on the non-contrast CT from PET/CT, so diagnostic contrast-enhanced CTs or MRIs obtained at baseline were segmented manually on axial slices (Fig. 2) by a radiologist specializing in hepatic malignancies (RK). These images were typically arterial-phase, but not all MRIs had contrast. Window/level was set to a liver default of 160/40 HU and then adjusted to maximize contrast. The diagnostic scan was then automatically rigidly registered to the CT of the ^90^Y PET/CT, fine manual adjustments were performed, and the lesion outlines were transformed to the ^90^Y PET/CT frame of reference (Fig. 2). In some cases, the radiologist adjusted lesion location manually on the ^90^Y PET/CT scan when mis-registration was evident. For example, when a lesion was partially outside the liver boundary visible on the CT of PET/CT, the radiologist performed a translation of the morphologically defined contour to lie within the liver at the appropriate location on the ^90^Y PET/CT. Lesion location was also adjusted when the morphologic contour was in close proximity to uptake on the ^90^Y PET, but did not coincide. This fine tuning of lesion location compensates for residual registration errors between the diagnostic morphologic scan and ^90^Y PET/CT; a single rigid registration is imperfect because the liver is deformable. In cases (*N* = 13) where a narrow window enabled tumor visualization on the non-contrast CT of the ^90^Y PET/CT, segmentation was performed directly on the non-contrast CT, thereby reducing registration errors.

A total of 85 tumors were initially segmented on the 21 scans. We excluded tumors < 2 cm^3^ because of limited spatial resolution of PET and sensitivity to mis-registration. An additional 5 tumors were excluded because they showed no PET uptake (defined as < 5 Gy AD), leaving 58 tumors across 17 scans for analysis.

### PET gradient-based segmentation

The commercial gradient-based method (PET Edge, MIM Inc., Cleveland, OH) was applied to ^90^Y PET images. It is a semi-automatic method requiring initial conditions determined through minimal user interaction that has been described previously [19]. Briefly, interaction consists of selecting a plane and then dragging out rays from the center of the lesion toward the edges (Fig. 1). Six rays extend along an orthogonal coordinate system as the user drags the ray radially away from the center. The rays define an ellipsoidal bounding volume for initial gradient detection. The user is allowed to change the angle of the rays by dragging, and the rays provide visual feedback showing an estimate of the gradient-determined edge. This is possible because the spatial gradients are interactively calculated along each ray and the length of the ray is restricted when a large spatial gradient, indicative of an edge, is estimated.

The gradient-based segmentations were performed by first localizing to the centroid of the MS. Although it is possible to union together multiple gradient-based segmentations to define a volume, we started at the center to avoid having to union volumes together. Thus, we used a single gradient-based segmentation operation for each lesion which potentially saves operator time, which is important clinically. The gradient-based segmentation was initiated from the center of the MS in the plane of maximum morphological extent and the user dragged the mouse so the rays were as close as possible to the boundary of the MS; we chose to use the gradient-based segmentation tool in this manner assuming there would be an accompanying morphologic scan where the tumor would be visible, but not segmented. Our method does not require MS segmentation, but it does require a registered scan showing the full morphological extent, which the MS segmentation provided for us. However, it should be noted that a CT is not required for the gradient-based segmentation. Intra-observer variability was assessed by generating a second gradient-based contour a month later by the same operator. Gradient-based segmentation on PET was performed by a medical physicist (JM). First and second gradient-based segmentations will be referred to as PS1 and PS2, respectively.

### Voxel-level AD and BED

Voxel-level dosimetry was performed with our dose planning nethod (DPM) Monte Carlo [23] code using the ^90^Y activity concentration values obtained directly from the PET image coupled with materials/densities obtained from the CT portion of PET/CT. The output of DPM is the dose-rate map, which was converted to an AD map accounting for ^90^Y physical decay only, because microspheres are permanently trapped. The voxel-level BED was calculated from differential absorbed dose volume histogram (DVH) using the reformulation of the linear quadratic model for radionuclide therapy [2]: $\mathrm{BE}{\mathrm{D}}_i={D}_i+\frac{D_i^2}{\frac{\alpha }{\beta }}\cdotp \left(\frac{\lambda }{\lambda +\mu}\right)$, where *D*_*i*_ is the absorbed dose at voxel i, *λ* is the physical decay constant (0.0108/h), *μ* is the cell repair constant (0.462/h), and *α*/*β* (10 Gy) is the ratio of radiosensitivity parameters typical for tumors. In addition to mean values, DVH metrics such as D90 (or BED90), which is the AD (or BED) delivered to 90% of the tumor volume, were also calculated.

### Statistical analysis

Concordance (ccc) and Pearson (*r*) correlation coefficients were estimated to quantify agreement and correlation between PS and MS measures (mean AD, D70, D90, mean BED, BED70, BED90). When estimating correlation, we used the average of the two PS realizations. We performed two PS segmentations to estimate intra-observer variability; and to use all data available, we decided to average the measures (mean, D70, D90, etc.) from the two PS realizations together for the concordance and correlation studies. To calculate 95% confidence intervals in the presence of possible correlated outcomes (between lesions within patient), we used a bootstrap approach with sampling at the patient level. To test for any mean difference in dose metrics between MS and PS regression models, regression models were fit with indicator variable for MS vs PS. To account for possible correlation between lesions within patient, we included random patient level intercept terms.

Spatial concordance between PS and MS and between the two PS realizations was assessed using the Dice similarity coefficient (DSC) and mean distance to agreement (MDA). DSC is defined as 2(VOI_1_ ∩ VOI_2_)/(VOI_1_ + VOI_2_), where VOI_1_ and VOI_2_ are the volumes from the two segmentations. A value of 1 represents perfect agreement and 0 indicates no spatial overlap [24]. MDA is the average distance between the surface of both contours with a value of 0 indicating perfect agreement [25]. Intra-observer variability between the two PS realizations was also assessed by calculating DSC and MDA. SAS software (v9.4) and R (v3.4.1) were used for statistical analysis.

## Results

### Phantom study

Qualitatively, there was good agreement between MS and PS as the segmentations nearly overlap (Fig. 1). Quantitative comparisons of the two segmentation methods and the two realizations of PS are given in Table 2. Figure 1a, b demonstrates the limitation of a fixed threshold segmentation of the 60 cm^3^ sphere with different TBR. A threshold of 18% provided the best volume estimate (61.8 cm^3^) for the MS volume (61.6 cm^3^) of the sphere in a cold background (Fig. 1a). A 32% threshold provided the best estimate (61.7 cm^3^) for the sphere in warm background (Fig. 1b). Using the 18% threshold with warm background overestimated (102.4 cm^3^) the MS volume, while using the 32% threshold in a cold background underestimated (50.8) the MS volume.

**Table 2:** Summary of phantom results for the two segmentation methods and the two PS realizations

	Sphere 8 cm^3^	Sphere 16 cm^3^	Ovoid 29 cm^3^	Sphere 60 cm^3^ (warm)	Sphere 60 cm^3^ (cold)
MS volume (cm^3^)	8.2	15.6	29.8	61.6	61.6
PS volume (cm^3^)^a^	9.4, 9.3	17.6, 17.4	28.7, 28.7	56.4, 56.4	61.7, 61.0
^b^PS vs MS					
DSC	0.87	0.86	0.86	0.93	0.97
MDA (mm)	1.20	1.47	1.40	1.20	0.63
ΔD_Mean_ (%)	− 4.7	− 2.4	1.8	3.5	0.7
ΔD70 (%)	− 7.7	− 4.2	3.1	7.4	1.1
ΔD90 (%)	− 7.5	0.8	4.5	8.3	3.0
PS1 vs PS2					
DSC	0.99	0.99	1.0	0.99	1.0
MDA (mm)	0.15	0.14	0.07	0.15	0.07
ΔD_Mean_ (%)	0.3	0.5	0.0	− 0.1	0.6
ΔD70 (%)	0.5	0.9	− 0.1	− 0.1	1.3
ΔD90(%)	0.4	1.5	− 0.1	− 0.4	2.2

^a^Realization 1 and 2

^b^PS realization with worst agreement tabulated. Δ represents the relative difference in the reported dose metric with respect to the MS value

### Patient study

#### Example segmentations

Figure 2 presents examples of MS and PS that include different levels of concordance. Figure 2a demonstrates a MS with only partial PET uptake; the CT defined contour was readily seen on contrast CT but it was not clearly visible on CT of PET/CT. There was concentrated uptake on the ^90^Y PET, but the MS appeared to not be fully perfused with microspheres, thus the PS yielded a much smaller volume and spatial concordance was poor as seen by DSC values indicated in the figure. In Fig. 2b, the large hypodense core of the lesion is clearly visible on both the diagnostic CT and CT of PET/CT. Although the microspheres were deposited along the lesion’s periphery, the PS was able to generate a single connected VOI that approximated the MS. Figure 2c shows a tumor with non-uniform PET uptake, but the PS still agreed well with the MS.

#### Spatial concordance and comparison of lesion AD/BED results

**Fig. 3:**
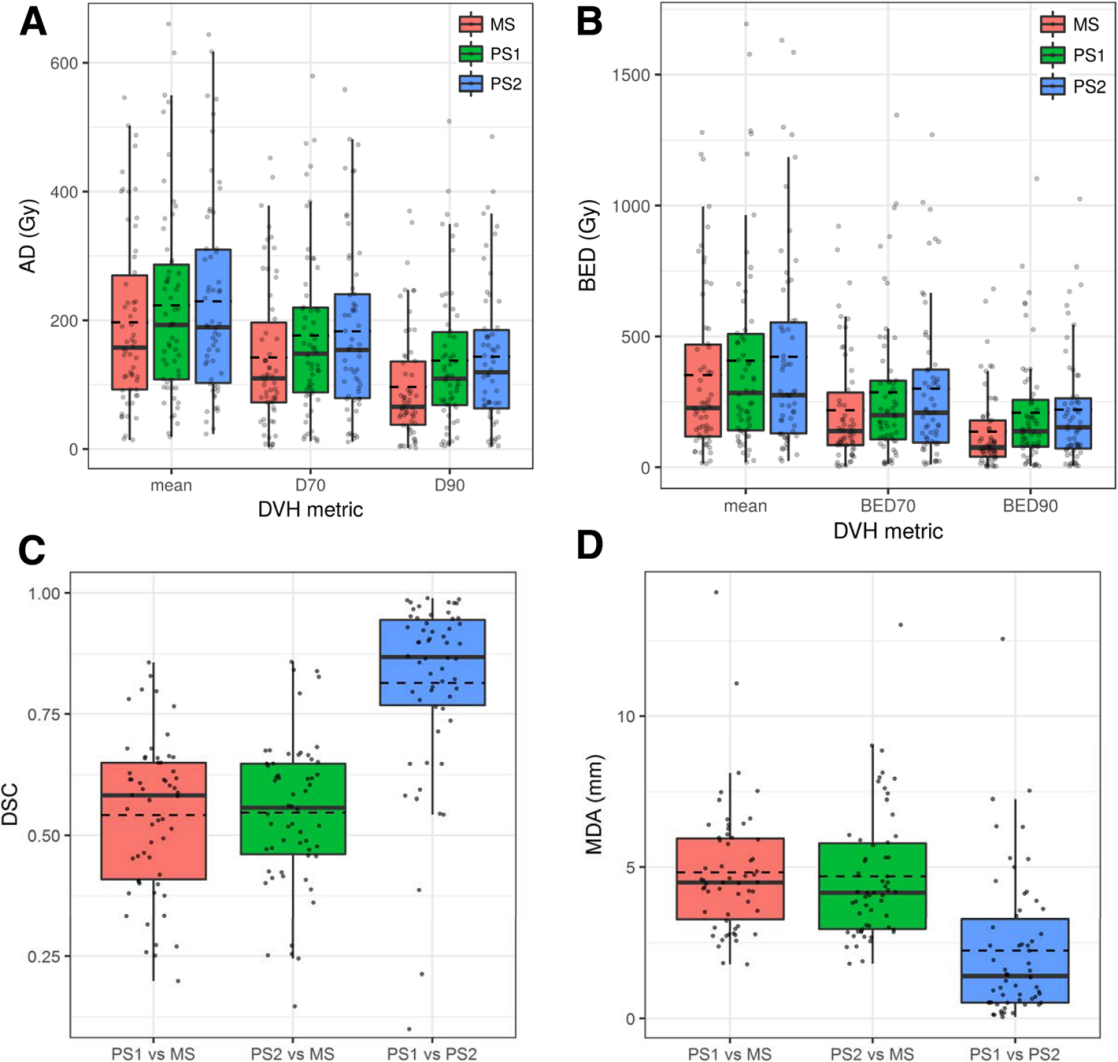
Boxplots summarizing the tumor AD (**a**) and BED (**b**) DVH metrics for MS, PS1, and PS2. Spatial concordance is summarized in boxplots using DSC (**c**) and MDA (**d**). For boxplots, the solid black line represents the median and dashed line represents the mean. The bottom and top of the box represent the 1st (Q1) and 3rd (Q3) quartile. Whiskers extend to the largest (or smallest) value within 1.5*(Q3 − Q1) + Q3 for largest (Q1 − 1.5 * Q3 − Q1) for smallest)

**Fig. 4:**
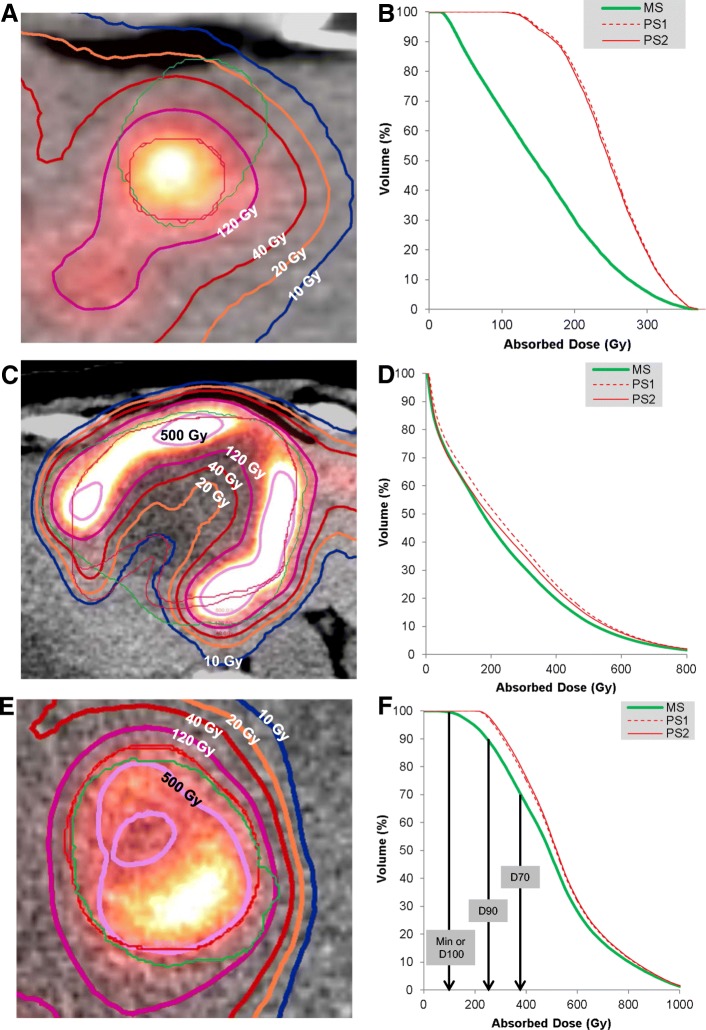
The absorbed isodose contours (thick lines) with morphologic (thin green lines) and PET-based (thin red lines) segmentations in (**a**), (**c**), and (**e**) with the corresponding lesion DVHs in (**b**), (**d**), and (**f**), respectively. These are the same lesions represented in Fig. 2

Boxplots of AD and BED DVH metrics for our population are shown in Fig. 3a, b. Spatial concordance between PS and MS, as well as PS1 and PS2, are shown in Fig. 3c, d. Absorbed isodose contours and cumulative DVHs corresponding to the tumors in Fig. 2 are presented in Fig. 4.

**Fig. 5:**
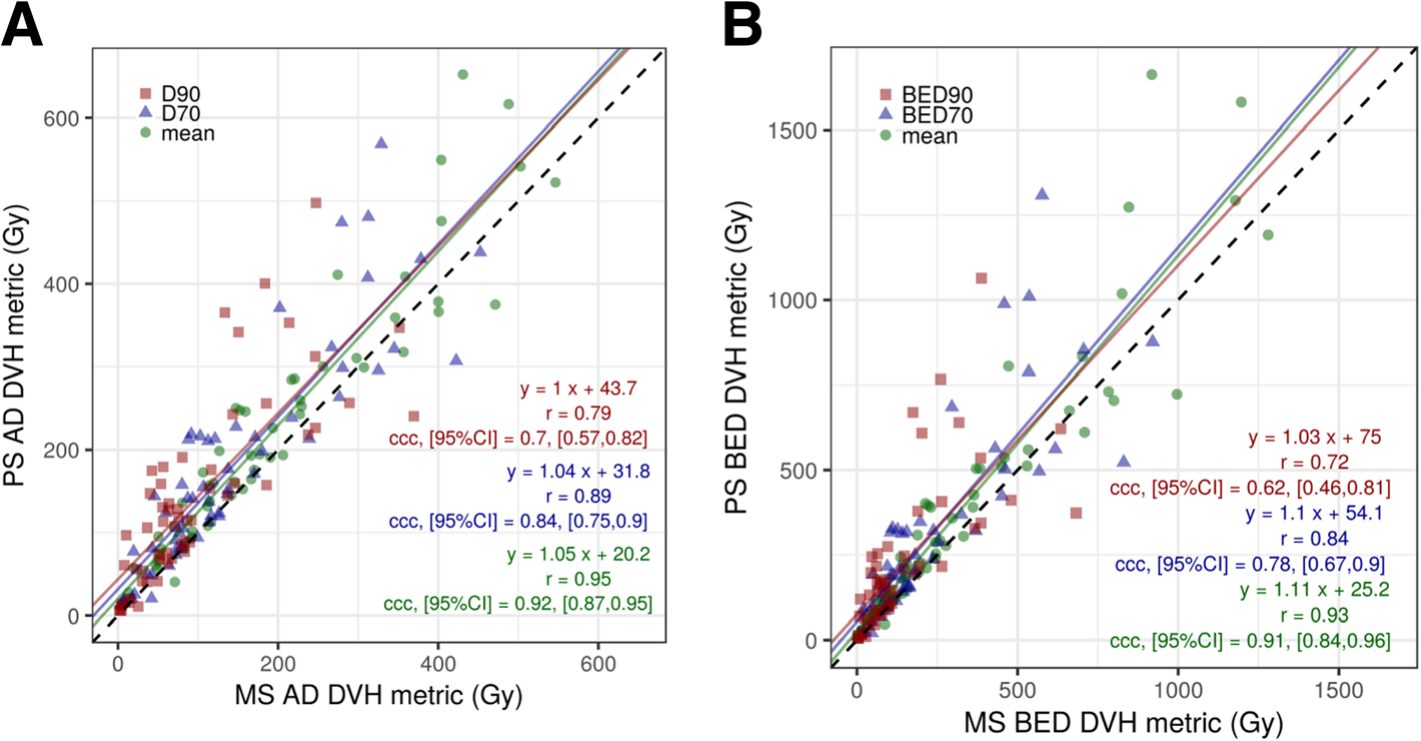
Tumor dose metrics for PET-based vs. morphologic segmentations for AD (**a**) and BED (**b**). Mean AD and BED (green circles), D70 and BED70 (blue triangles), D90 and BED90 (red squares). The dashed line is the line of equivalence

**Fig. 6:**
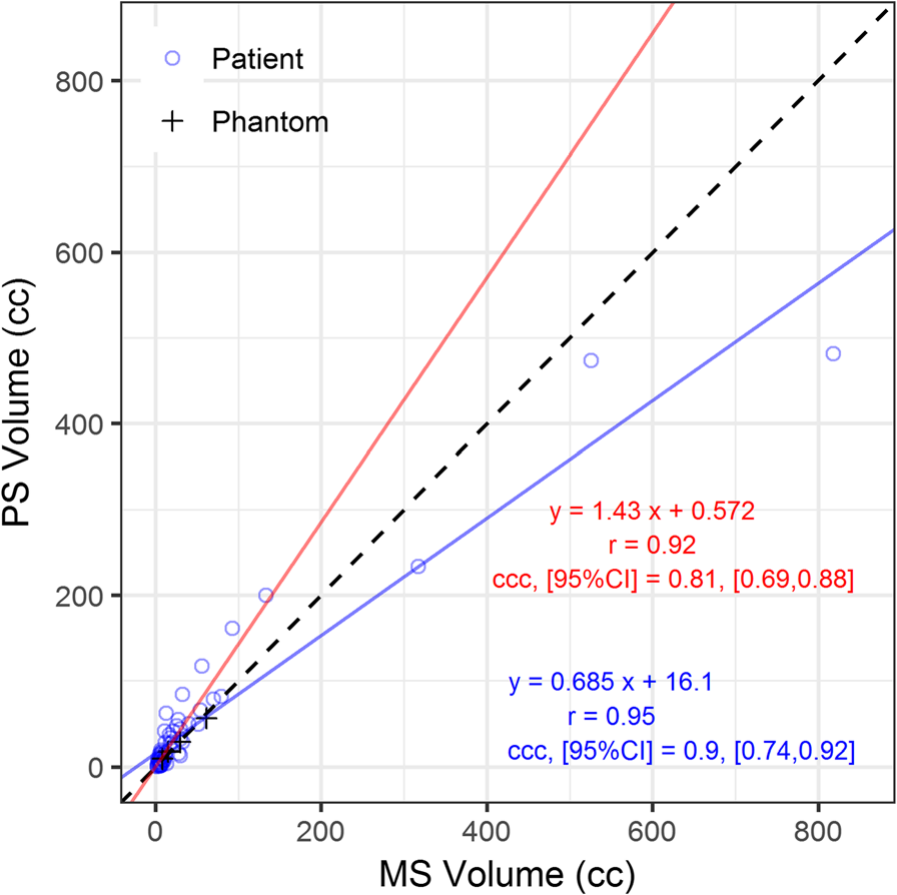
Plot comparing PS volumes with MS volumes for 58 tumors. Blue fit line includes all lesions and the red fit line excludes 3 lesions > 200 cm^3^. The dashed line is the line of equivalence, and the black crosses represent our phantom data

Because Fig. 3 shows low intra-observer variability for PS, when estimating correlation (Figs. 5 and 6, the average of the two PS realizations was used. The AD/BED metrics calculated using PS are plotted against the respective quantity calculated from the MS in Fig. 5. PS volumes are plotted against MS volumes in Fig. 6. Excluding three large volumes (> 200 cm^3^ on CT) with no central uptake (similar to lesion of Fig. 2b) changed the fit substantially. Additional details on volume and DVH metrics are tabulated in Tables 3 and 4.

**Table 3:** Summary of volume and AD/BED metrics for morphologic and PET-based segmentation

	MS	PS	PS - MS [95% CI]
Volume (cm^3^)	48 ± 130 [2,6,11,30,818]	49 ± 94 [0.3,6,16,47,482]	0.8 [− 6.2, 7.9] *p* = 0.84
D_mean_ (Gy)	197 ± 138 [14,92,158,270,546]	227 ± 153 [21,107,192,300,652]	30 [20, 39] *p* < 0.0001
D70 (Gy)	142 ± 113 [3,72,110,197,452]	180 ± 132 [13,82,145,226,569]	38 [26, 49] *p* < 0.0001
D90 (Gy)	96 ± 88 [2,37,65,136,370]	140 ± 111 [6,64,115,179,498]	44 [31, 57] *p* < 0.0001
BED_mean_ (Gy)	352 ± 325 [14,116,226,469,1280]	415 ± 385 [22,137,282,530,1663]	63 [35, 90] *p* < 0.0001
BED70 (Gy)	217 ± 218 [3,84,137,285,920]	293 ± 285 [13,97,194,342,1309]	76 [47, 105] *p* < 0.0001
BED90 (Gy)	135 ± 152 [2,40,75,178,682]	214 ± 217 [6,73,145,252,1064]	79 [50, 107] *p* < 0.0001

Data in columns 1 and 2 are mean ± stdev [min, 1st quartile, median, 3rd quartile, max]

**Table 4:** Difference in volume and AD/BED metrics at the lesion level when comparing the two segmentation methods and the two realizations of PET-based segmentation

	PS vs MS	PS1 vs PS2
ΔVolume (cm^3^)	0.8 ± 51 [− 336, − 2, 3, 12, 69]	5 ± 40 [− 78, − 1, 0,4256]
Absorbed Dose		
ΔMean (Gy)	30 ± 50 [− 96, 1, 17, 45, 222]	− 6 ± 23 [− 103, − 13, − 0,3,37]
ΔD70 (Gy)	38 ± 61 [− 115, 1, 18, 57, 240]	− 7 ± 23 [− 85, − 10, − 0,4,40]
ΔD90(Gy)	44 ± 68 [−129, 3, 24, 70, 250]	−6 ± 23 [− 84, − 7, 0,3,51]
BED		
ΔMean (Gy)	63 ± 142 [− 273, 2, 30, 80, 745]	− 14.7 + − 55 [− 266, − 22, − 1, 7, 67]
ΔBED70(Gy)	76 ± 155 [− 308, 2, 29, 113, 733]	− 13.8 + − 49 [− 197, − 16, − 1, 6, 75]
ΔBED90 (Gy)	79 ± 152 [− 309, 3, 30, 107, 677]	− 11.6 + − 42 [− 170, − 17, 0, 5, 78]
ΔVolume (%)	44 ± 100 [− 85, − 27, 18, 99, 382]	54 ± 253 [− 75, − 10, 1, 25, 1779]
Absorbed Dose		
ΔMean (%)	24 ± 36 [− 43, 2, 14, 36, 193]	− 2.5 ± 11 [− 39, − 7, − 0, 2, 21]
ΔD70 (%)	49 ± 72 [− 51, 2, 24, 75, 318]	− 1.9 ± 16 [− 48, − 9, − 0, 4, 39]
ΔD90(%)	93 ± 150 [− 58, 8, 46, 125, 832]	− 0.9 ± 21 [− 52, − 10, 0, 4, 72]
BED		
ΔMean (%)	28 ± 42 [− 46, 2, 17, 46, 221]	− 3.0 + − 13 [− 45, − 8, − 0,3,25]
ΔBED70(%)	62 ± 85 [− 53, 2, 31, 105, 345]	− 2.3 + − 18 [− 54, − 12, − 0,5,42]
ΔBED90(%)	117 ± 183 [− 60, 10, 54, 174, 1026]	− 1.2 + − 24 [− 59, − 13, 0,4,89]

The results are listed as mean ± stdev [min, 1st quartile, median, 3rd quartile, max]. The top half of the table represents absolute differences and the bottom half represents relative differences

## Discussion

The phantom experiments, performed under the clinically realistic noise conditions of ^90^Y PET, demonstrated high accuracy in lesion volumes (within 15%), AD metrics (within 8%), and high spatial concordance (DSC > 0.86, MDA < 1.5 mm) for PS vs MS (Table 2). When comparing the two PS realizations for the phantoms, the intra-observer variability was low (DSC > 0.99, MDA < 0.2 mm, difference in AD metrics < 1.5%).

In patient studies, for tumors < 200 cm^3^, the PET-based method tended to generate larger volumes than the corresponding morphologically driven one (Fig. 6). A possible explanation for the larger PET-based volumes stems from the non-uniformity of the ^90^Y PET including higher noise levels and respiratory motion leading to spatial spreading of the activity distribution. The AD and BED metrics for PET-based segmentation, on average, are larger than those corresponding to the MS VOIs (Fig. 3a, b). This appears counterintuitive because the PET-based method in general also had larger volumes. However, the gradient-based method tends to “seek” out or encompass the activity, thus the preferential localization of activity partially compensates for differences in volume. In addition, residual registration errors, although minimized, may play a role in decreasing the activity contained in MS. An example of the larger PS relative to MS is included as Additional file 1 Supplemental Figure 1. Spatial concordance of the two methods (Fig. 3c, d) is worse than in the phantom studies; however, 75% of tumors still had MDA within 5.8 mm, which is less than 1.5 times our PET voxel length. The three PS1 vs PS2 outliers in Fig. 3c were further investigated and showed large differences in volume (0.07 vs 14.8 cm^3^, 1.1 vs 9.7 cm^3^, 0.4 vs 1.5 cm^3^). The MDA outlier for PS1 vs PS2 (Fig. 3d) was also one of these three. The first PS was localized on a relatively intense uptake within the MS VOI, while the second PS encompassed a volume slightly larger than the MS. Thus, these were sensitive to initial conditions of the gradient-based segmentation, and additional investigation is needed in the future on this topic. The degradation of spatial concordance in patient measurements is not surprising considering potential biological changes in vasculature and flow dynamics between baseline and post-therapy imaging, residual registration errors, respiratory motion, and liver deformations between scans.

For patient AD metrics, the best correlation and concordance between segmentation methods was found for the mean absorbed dose (*r* = 0.94, ccc = 0.92) (Fig. 5). As the AD metric approached the minimum dose (mean ➔ D70 ➔ D90), both the correlation and concordance worsened (*r* = 0.77, ccc = 0.70). BED followed a similar trend. A possible reason for worse concordance with coverage metrics is that as discussed above, the gradient-based method “seeks” out the activity and localizes and conforms over the uptake, whereas the shape of the morphologic segmentation is independent of the activity distribution. This leads to differences in spatial overlap between contours in the “lower” dose regions while the “higher” dose regions tended to overlap (Fig. 4a). The DVHs that are presented also demonstrate this effect; the differences between MS and PS curves increase for two of the three example cases as one approaches the minimum dose to the VOI. Another potential reason for differences could be due to respiratory motion; the ^90^Y signal will spread out spatially over several respiratory cycles leading to a larger “low” dose region encompassed by the PS, whereas the MS was based on MRI or CT from diagnostic studies or the CT component from the free-breathing PET/CT when lesions were visible on CT.

In terms of intra-observer variability of PS in the patient studies, the average DSC of 0.81 and MDA of 2.2 mm (Fig. 3c, d) are worse than the worst in-phantom measurements; however, 75% of lesions had MDA within 3.3 mm, which is less than a single PET voxel length. A potential reason for differences includes non-uniform uptake in less well-defined (no sharp fall off) geometries in the case of patients when compared with phantoms. Furthermore, in theory, the PET gradient-based method is robust to non-uniform distributions, but from a practical point of view, the version studied still requires initial conditions specified by the user which may not have been reproduced in cases where the plane of maximum extent was not clear or in cases where the feedback for edge detection differed because a different ray angle was selected. Such cases can be seen in PS1 vs PS2 in Fig. 3c, and PS1 vs PS2 in Fig. 3d. Although there was variability between the PS realizations AD/BED metrics with standard deviations ranging from 11 to 24%, the effect on average was ≤ 3% (Table 4).

**Fig. 7:**
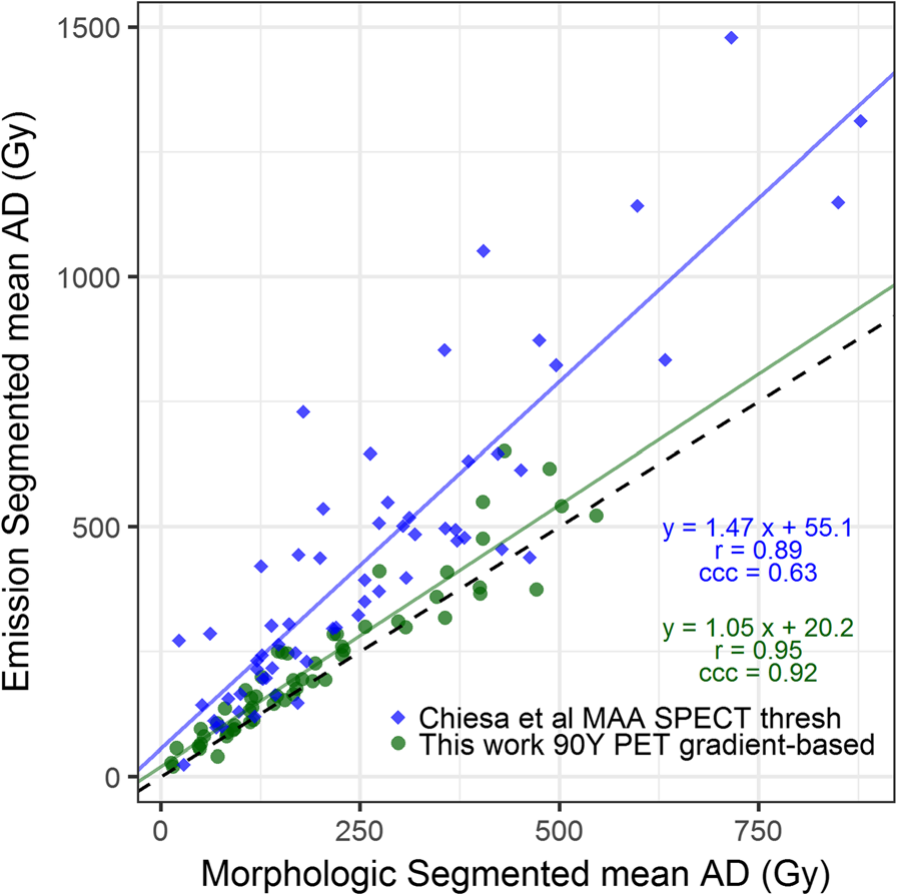
Showing our PS vs MS for mean tumor AD alongside ^99m^TcMAA SPECT threshold-based segmentation vs MS for mean tumor AD data from Chiesa et al. [14]

There are no studies, to the best of our knowledge, that have evaluated lesion segmentation on post-therapy ^90^Y PET/CT. However, Chiesa et al. [14] compared thresholding on the pre-therapy ^99m^TcMAA SPECT with CT-defined volumes for 60 HCC lesions; they found that the median of the mean AD for tumor response was 522 Gy for thresholding and 339 Gy for CT-based segmentation. In Fig. 7, we plot the mean ADs of our PS vs MS alongside Chiesa et al.’s results for threshold-based segmentation vs MS to demonstrate the differences in concordance achieved in two studies. Although it is difficult to make a direct comparison between Chiesa et al. and our study (HCC vs multiple diseases, thresholding vs gradient, and ^99m^TcMAA SPECT vs ^90^Y PET), the data suggests that gradient-based segmentation on ^90^Y PET is more representative of the morphological delineated tumor volume than the thresholded ^99m^TcMAA VOI. A patient example of this is that hypovascular cores were included when using PS (for example, see Fig. 2b), whereas with thresholding the core would not be included. It is unclear whether or not to include such cores when reporting tumor dosimetry. However, it is possible that our method of localizing to the MS and initializing the gradient-based algorithm’s ray to match the MS may have improved agreement. We believe that from a practical point of view, this process is very similar to registering a diagnostic CT or MRI to the ^90^Y PET/CT and then initializing to the boundary seen on the fused diagnostic scan that has not been segmented.

One of the advantages of gradient-based segmentation is that it can handle non-uniform distributions and different TBRs. A phantom example of this is shown in Fig. 1a, b, where the same sphere in cold and warm background was segmented by morphological, gradient-based, and thresholding. The optimal threshold of 18% in the cold background overestimated the MS in the warm background by approximately 66%, while the optimal threshold of 32% in the warm background underestimated the MS volume in cold background by approximately 17%. Gradient-based segmentation was within 8.5% of the MS volume. Given these observations in our phantom data and the strong dependence of the optimal threshold on TBR, lesion size, and other factors discussed in a recent AAPM report [16] and past review [26], threshold-based segmentation was not pursued for the patient studies.

Strengths of this study include segmentation on post-therapy ^90^Y PET/CT potentially for the first time, validation on clinically realistic phantom studies, using commercially available clinical segmentation tools, and reporting multiple AD/BED DVH metrics, which have been used in previous dose-response studies [13, 27]. There are more advanced segmentation methods for PET than the gradient-based method evaluated here, but most of these are in the research setting and not available in commercial clinical software [16]. Finite spatial resolution and noise are inherent limitations to all segmentation methods. Based on our clinically realistic phantom data, the gradient-based segmentation is highly accurate for our reconstruction parameters, including 5 mm FWHM post-reconstruction blurring. However, these inherent limitations may hinder the accurate localization and identification of gradients, and one method of compensating for these limitations involves deconvolution and bilateral filtering that preserves edges [16, 17]. Limitations include the small number of patients, the mix of primary and metastatic hepatic malignancies, and potential impact of mis-registrations. The impact of registration errors was offset by restricting analysis to lesions > 2 cm^3^. The use of deformable image registration in the liver was beyond the scope of this study, but it should be investigated in future work. In addition, inter-observer variability for both segmentation methods and an estimate of intra-observer variation for MS should be investigated. It was not the purpose of this work to determine if one method is clinically superior, but rather to estimate the differences in reporting AD/BED between the gradient-based and morphological segmentation.

There was not a gold standard in this work. One potential “truth” for clinical segmentation would require excising lesions and liver segments followed by sectioning and histo/pathology analysis to identify lesion boundaries. Uncertainties including registration, deformation, and interval between imaging and excision would still exist, so this was not pursued. In addition, it is difficult to acquire such data in routine clinical workflows due to extra resources required.

^90^Y glass microspheres are delivered based on blood flow and become physically trapped, not metabolized. They are not a biochemical or molecular-based therapy. Segmenting lesions solely on activity from the ^90^Y PET assumes that microspheres came to rest within a lesion; of course, this may not be true. Consequently, it is prudent to perform segmentation with additional information, such as a contrast CT or MR to help localize the lesions. This is consistent with the PS methodology described in this work.

Correlation between lesion AD and response is beyond the scope of the current study and will be undertaken in the future. Several studies have shown correlations between tumor response and mean absorbed dose or mean biological effective dose for ^90^Y microspheres [14, 28–30]. There may be additional value to calculate macroscopic absorbed dose heterogeneity (e.g., incomplete perfusion, necrotic cores). Using $\frac{\alpha }{\beta }=10\ \mathrm{Gy}$ and *α* = 0.004\Gy [14], we found strong correlation for EUD vs mean absorbed dose (*r* > 0.98) and equivalent uniform BED (EUBED) vs mean BED (*r* > 0.95) for both MS and PS using ^90^Y microsphere PET/CT, thus we did not report EUD or EUBED. For the current absorbed dose levels and value of alpha presented, the EUD and EUBED can be well approximated by a linear function, due to expansion of exponential. This leads to the EUD and EUBED calculating the means, which explains the high correlation between the mean absorbed dose and EUD. The goal of a segmentation method on ^90^Y microsphere PET is to report AD/BED DVH metrics that will reliably predict tumor control and normal tissue complications. This work directly addressed differences in DVH metrics due to segmentation methods applied to lesions, and the results may aid in the long-term harmonization of reporting AD/BED metrics across institutions.

## Conclusion

Phantom studies showed accurate and robust results for ^90^Y PET-gradient-based segmentation that is practical to use in the clinic. Quantitative comparisons with morphologically driven lesion segmentations in patient studies showed high concordance for mean AD and BED while DVH coverage metrics such as D70 and D90 were less concordant between the two segmentation methods. Estimated differences in reported AD/BED metrics due to segmentation method will be useful for interpreting RE dosimetry results in the literature including tumor response data. These differences highlight the need for the RE community to standardize segmentation methods for reporting of lesion dosimetry on post-therapy ^90^Y PET.

## Additional file

Additional file 1: Supplemental Figure 1: Example showing PS extension beyond MS. (PDF 120 kb)

## Abbreviations

^18^F: Fluorine-18
3D: Three dimensional
^90^Y: Yttrium-90
95%CI: 95% confidence interval
^99m^Tc: Technetium-99 m
AAPM: American Association of Physicists in Medicine
AD: Absorbed dose
BED: Biological effective dose
BED70: The minimum biological effective dose delivered to 70% of the volume determined from a cumulative dose-volume histogram.
BED90: The minimum biological effective dose delivered to 90% of the volume determined from a cumulative dose-volume histogram.
ccc: Concordance correlation coefficient
CT: Computed tomography
D70: The minimum absorbed dose delivered to 70% of the volume determined from a cumulative dose-volume histogram.
D90: The minimum absorbed dose delivered to 90% of the volume determined from a cumulative dose-volume histogram.
DPM: Dose planning method
DSC: Dice similarity coefficient
DVH: Dose volume histogram
FDG: Fluorodeoxyglucose
HCC: Hepatocellular carcinoma
MAA: Macroaggregated albumin
MDA: Mean distance to agreement
MRI: Magnetic resonance imaging
MS: Morphologic driven segmentation
OSEM: Ordered subset expectation maximization
PET: Positron emission tomography
PS: 90Y PET gradient-based segmentation
PS1: The first realization of gradient-based segmentation
PS2: The second realization of gradient-based segmentation
Q1: First quartile
Q3: Third quartile
r: Pearson’s correlation coefficient
RE: Radioembolization
SPECT: Single photon emission computed tomography
TBR: Tumor-to-background ratio
VOI: Volume of interest
*α*/*β*: Radiosensitivity in BED equation
*λ*: Physical decay rate constant in BED equation
*μ*: Cell repair rate constant in BED equation
