# Supplementary material for: Impact of 90Y PET gradient-based tumor segmentation on voxel-level dosimetry in liver radioembolization
Source: EJNMMI Phys. 2018 Nov 30;5:31. doi: 10.1186/s40658-018-0230-y (PMC6265358; doi:10.1186/s40658-018-0230-y)
Supplement: Supplementary file 1 — Supplemental Figure 1: Example showing PS extension beyond MS. (PDF 120 kb) [file 40658_2018_230_MOESM1_ESM.pdf]

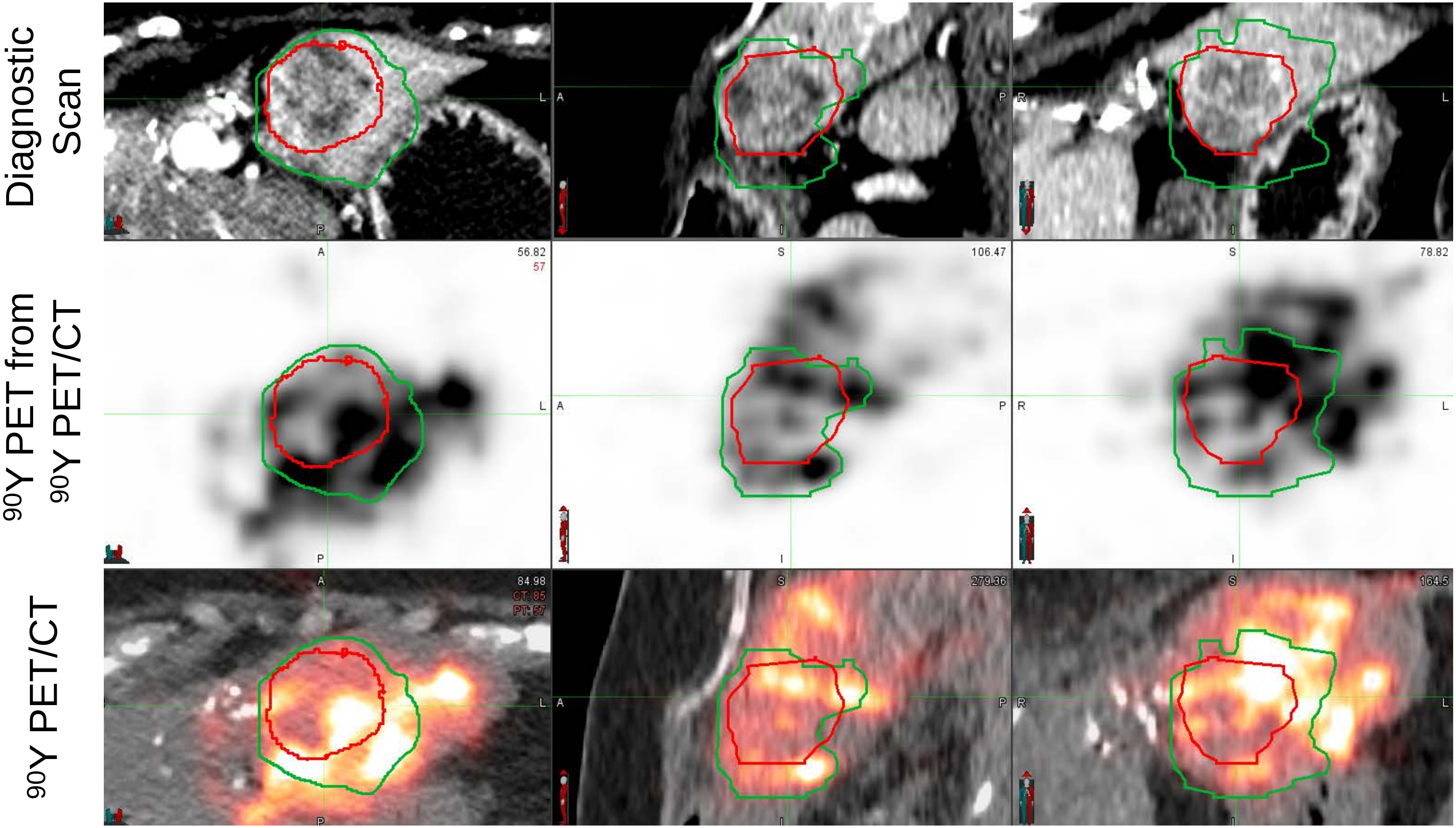

Patient 8, lesion 3 illustrates the PS (green contour) extending beyond the MS (red contour) and encompassing surrounding activity. This can be seen clearly on the 90Y PET images in the central row. The PS has a larger volume (58 cc) than MS (21 cc), but also contains more activity so the mean absorbed doses reported are similar. In this example the mean absorbed dose was 82.7 Gy for MS, and 83.2 Gy for PS. The DSC was 0.52 and MDA was 5.6 mm.
